# *OsSTS*, a Novel Allele of *Mitogen-Activated Protein Kinase Kinase 4* (*OsMKK4*), Controls Grain Size and Salt Tolerance in Rice

**DOI:** 10.1186/s12284-023-00663-y

**Published:** 2023-10-24

**Authors:** Jianguo Liu, Lan Shen, Longbiao Guo, Guangheng Zhang, Zhenyu Gao, Li Zhu, Jiang Hu, Guojun Dong, Deyong Ren, Qiang Zhang, Qing Li, Dali Zeng, Changjie Yan, Qian Qian

**Affiliations:** 1 Rice Research Institute, Shenyang Agricultural University, Shenyang, 110866 China; 2 State Key Laboratory of Rice Biology and Breeding, China National Rice Research Institute, Chinese Academy of Agricultural Sciences, Hangzhou, 311401 China; 3 Jiangsu Co‐Innovation Center for Modern Production Technology of Grain Crops/Agricultural College, Yangzhou University, Yangzhou, 225009 China; 4 The Key Laboratory for Quality Improvement of Agricultural Products of Zhejiang Province, College of Advanced Agricultural Sciences, Zhejiang A & F University, Hangzhou, 311300 China

**Keywords:** Rice, OsSTS/OsMKK4, Salt stress, ROS, ABA

## Abstract

Soil salinization is one of the most common abiotic stresses of rice, which seriously affects the normal growth of rice. Breeding salt-tolerant varieties have become one of the important ways to ensure food security and sustainable agricultural development. However, the mechanisms underlying salt tolerance control still need to be clarified. In this study, we identified a mutant, termed *salt-tolerant and small grains*(*sts*), with salt tolerance and small grains. Gene cloning and physiological and biochemical experiments reveal that *sts* is a novel mutant allele of *Mitogen-activated protein Kinase Kinase 4* (*OsMKK4*), which controls the grain size, and has recently been found to be related to salt tolerance in rice. Functional analysis showed that *OsSTS* is constitutively expressed throughout the tissue, and its proteins are localized to the nucleus, cell membrane, and cytoplasm. It was found that the loss of *OsSTS* function enhanced the salt tolerance of rice seedlings, and further studies showed that the loss of *OsSTS* function increased the ROS clearance rate of rice seedlings, independent of ionic toxicity. In order to explore the salt tolerance mechanism of *sts*, we found that the salt tolerance of *sts* is also regulated by ABA through high-throughput mRNA sequencing. Salt and ABA treatment showed that ABA might alleviate the inhibitory effect of salt stress on root length in *sts*. These results revealed new functions of grain size gene *OsMKK4*, expanded new research ideas related to salt tolerance mechanism and hormone regulation network, and provided a theoretical basis for salt-tolerant rice breeding.

**Supplementary Information:**

The online version contains supplementary material available at 10.1186/s12284-023-00663-y.

## Background

Soil salinization has become an increasingly serious problem in global agriculture. It is also one of the biggest limiting factors in agricultural production, which seriously affects the normal growth of crops and restricts crop yield and quality potential (van Zelm et al. 2020; Zhu 2016; Zhao et al. 2021; Munns and Tester 2008; Ren et al. 2023). Rice (*Oryza sativa*) feeds about half of the world’s population and is one of the most important salt-sensitive cereal crops (Wang et al. 2012; Jia et al. 2022; Zhang et al. 2021). To survive on saline soil, rice has evolved a complicated adaptive mechanism by multiple genes and pathways (Ganie et al. 2019; Yang and Guo 2018; Ponce et al. 2021). Exploring salt-tolerant genes and further understanding the regulatory mechanisms of salt stress responses in rice has important implications for breeding salt-tolerant rice varieties and global food security but remains a great challenge.

Mining salt-tolerant genes and breeding salt-tolerant varieties have become one of the most important ways to ensure food security and sustainable agricultural development (van Zelm et al. 2020). So far, many salt-stress-related genes in plants have been cloned and studied in depth. *AtNHX1* and its direct homologs are overexpressed in plant species such as *Arabidopsis thaliana* and tomato (*Solanum lycopersicum*) or rice, resulting in increased plant salinity tolerance (Zhang and Blumwald 2001; Apse et al. 1999). Many salt-tolerant genes in rice have been reported and even applied in production practice, which includes *SOS1* (the salt overly sensitive), *SOS2*, *SOS3*, and *OsHKT1*(high-affinity potassium (K^+^) transporter) (Zhu 2016; Chu et al. 2021). It has been found that plants, in response to extracellular abiotic stress, activate a complex intercellular signaling cascade that regulates physiological and biochemical changes. The Mitogen-activated protein kinase (MAPK) cascades consist of three layers of sequentially phosphorylating and activating protein kinases, including MAPK kinase kinases (MAPKKKs), MAPK kinases (MAPKKs), and MAPKs, which are highly conserved eukaryotic signaling modules acting downstream of the receptors in transducing extracellular stimuli into cellular responses (Wang et al. 2022b, 2014; Jia et al. 2022; Liao et al. 2021). There are 17 MAPKs, 8 MAPKKs, and 75 MAPKKKs in the rice genome, which play crucial roles in the transduction of environmental and developmental signals, and response to various stresses (Wankhede et al. 2013; Singh et al. 2014; Hamel et al. 2006; Jagodzik et al. 2018). Previous studies have shown that some MAPK genes play an important role in salt stress, and *OsMKK1* positively regulates rice salt tolerance through phosphorylated the downstream substrate OsMPK4 under salt stress (Wang et al. 2014; Zaidi et al. 2016); OsMKK4, whose kinase activity was induced by salinity, activates the kinase activity of OsMPK6 (Kumar et al. 2008; Shen et al. 2010; Pitzschke et al. 2014). *OsMAPK3* and *OsMAPK33* play an important role in the presence of salt stress (Lee et al. 2011; Schmidt et al. 2013; Zhang et al. 2018). In addition, plant hormones are known to affect signaling through MAPK cascades, mainly including auxin (AUX), abscisic acid (ABA), ethylene (ETH), brassinosteroids (BR), etc. (Jagodzik et al. 2018).

Among the above MAPK-related hormones, salt stress could induce the expression of ABA biosynthetic genes (NINE-CISEPOXYCAROTENOID DIOXYGENASEs (NCEDs) and ABA DEFICIENTs (ABAs) in specific vascular tissues (Julkowska and Testerink 2015). During salt stress, cellular ABA accumulating is perceived by ABA receptors causing ABA to bind to PYRABACTIN RESISTANCE‐LIKE (PYL) receptors that in turn bind to and inactivate PROTEIN PHOSPHATASE 2C (PP2C), which leads to the release of activated SnRK2 phosphorylates downstream targets, then triggering ABA-induced physiological and molecular responses (Ullah et al. 2020). Cell wall cellulose synthase-like D4 protein (OsCSLD4) can enhance rice ABA synthesis gene expression, increase ABA content and improve salt tolerance in rice (Zhao et al. 2022). Overexpression of *OsNAC2* can enhance salt tolerance in rice through ABA-mediated pathways (Jiang et al. 2019). In addition, OPEN STOMATA 1 (OST1) (also known as the best classic target of PP2Cs), which regulates stomatal opening, plays a major role in ABA signaling through the phosphorylation of downstream targets, ultimately triggering the production of apoplast reactive oxygen species (ROS) causing stomatal closure (Pei et al. 2000; Postiglione and Muday 2020; Chen et al. 2021; Han et al. 2019). Guard cells maintain ROS homeostasis during ABA signaling through antioxidant enzymes containing catalase (CAT), superoxide dismutase (SOD), etc. (Chen and Gallie 2004; Jannat et al. 2011; Miao et al. 2006; Tiew et al. 2015).

Reactive oxygen species can be viewed as a developmental and hormonal response signal (Huang et al. 2019; Mittler 2017), and MAPKs have also been implicated in ABA signaling and are also downstream targets of ROS (de Zelicourt et al. 2016; Postiglione and Muday 2020; Danquah et al. 2014). It has been introduced in *Arabidopsis*, the ABA core signaling pathway rapidly senses and responds to environmental changes under salt stress by mediating several rapid responses, including gene regulation, stomatal closure, and reduction of excess ROS levels (de Zelicourt et al. 2016; Postiglione and Muday 2020). However, the molecular mechanism of this process and its application are rarely mentioned in rice. In this study, we characterized a mutant exhibiting small grains and a strong salt-tolerant characteristic, named *salt-tolerant and small grains* (*sts*). *OsSTS* encoded mitogen-activated protein kinase kinase 4 and turned out to be a new allele of *OsMKK4*. We next identified the mutated gene and found that the small grains and a strong salt-tolerant characteristic of the *sts* mutant were caused by a frameshift mutation of the *sts* gene. Under salt stress, the destruction of *OsSTS* increased the survival rate, decreased the expression level of ROS, and promoted the expression levels of ABA biosynthesis genes and ABA signaling genes of rice seedlings. Salt and ABA treatments showed that ABA alleviated the inhibitory effect of salt stress on *sts* root length; that is, *OsSTS* improved the salt tolerance of rice by regulating ABA content under salt stress. In conclusion, the functional analysis of the *OsSTS* gene improves the mechanism of gene function and provides a theoretical basis for the creation of new rice germplasm with high yield and salt tolerance.

## Results

### Phenotypic Characteristics of the Rice sts Mutant

**Fig. 1:**
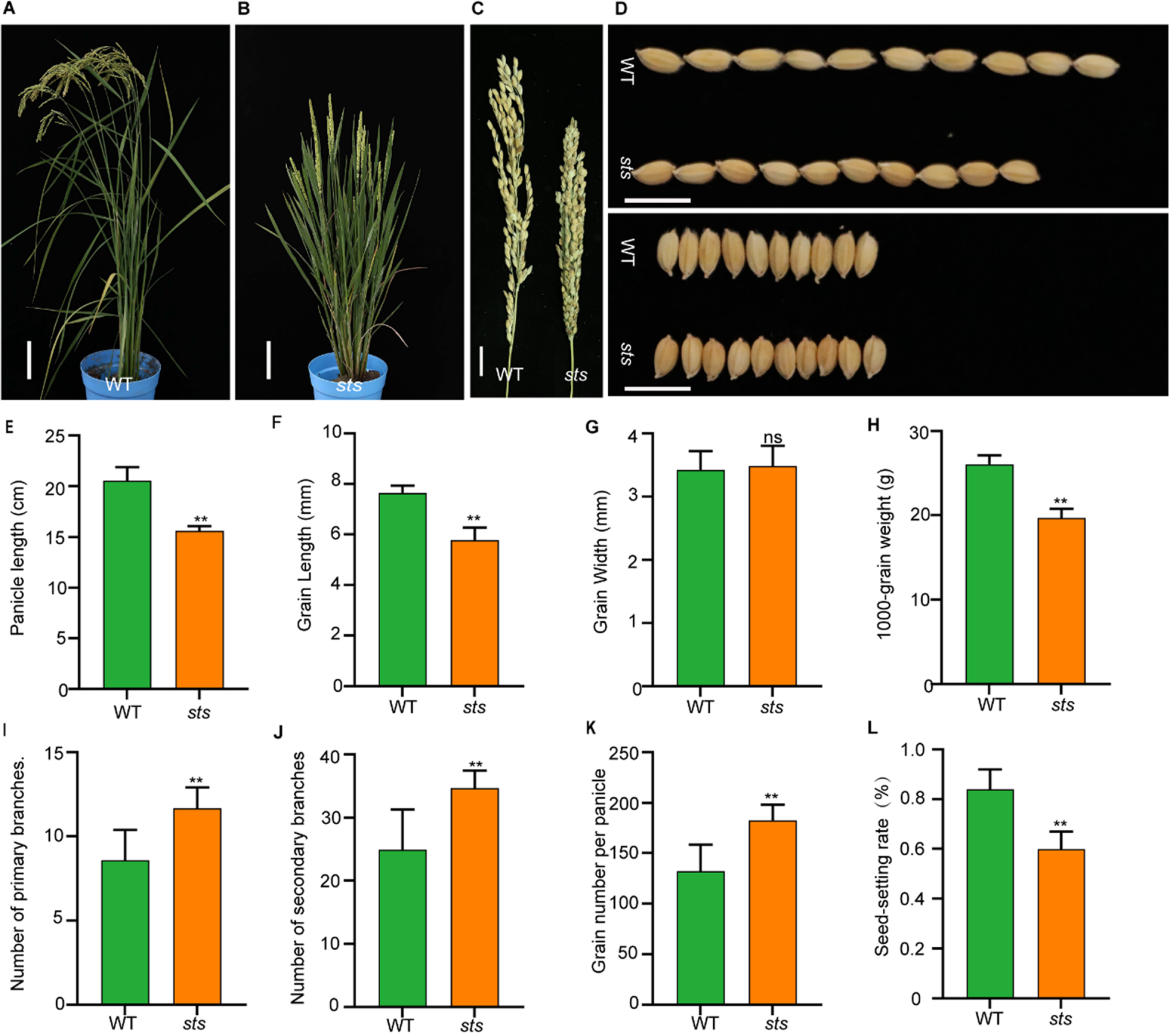
Phenotypic characterization of the *sts* mutant. **A, B** Phenotypes of wild-type (ZH11) and mutant (*sts*) at the adult stage, bar = 10 cm. **C** Panicles of WT and *sts*, bar = 2 cm. **D** Morphology of grain length and grain width in WT and *sts*, (bar = 1 cm). **E-L** Statistical data of the panicle length, grain length, grain width, 1000-grain weight, number of primary branches, number of secondary branches, grain number per panicle, and seed-setting rate in the WT and *sts*. Data are shown as mean ± SD (n = 15). **P < 0.01, Student’s t-test

The *sts* mutant was successfully obtained from mutagenesis of the japonica cultivar Zhonghua11 (ZH11) irradiated with ^60^Co-γ. At the mature stage, the phenotypic analysis indicated that the *sts* mutant exhibited shorter plant and panicle heights, and more erect panicles and leaves, compared with that of the wild type (Fig. 1A–C, E). Compared with the wild type, the seed setting rate of *sts* was significantly reduced, while the primary panicle branch number, secondary panicle branch number, and grain number per panicle of *sts* were markedly increased (Fig. 1I–L). As is shown, the *sts* mutant had significantly smaller grains than the wild type (Fig. 1D). Combined with the statistical results, the grain lengths of the *sts* mutant were significantly shorter than that of the wild type, while the grain widths of *sts* were not significantly different from that of the wild type (Fig. 1F, G). We also found that the 1000-grain weights of *sts* were markedly decreased compared with that of the wild type (Fig. 1H). These results implied that *OsSTS* might affect grain size and weight in rice.

### Map-Based Cloning of OsSTS, an Allele That Encodes a Mitogen-Activated Protein Kinase 4

**Fig. 2:**
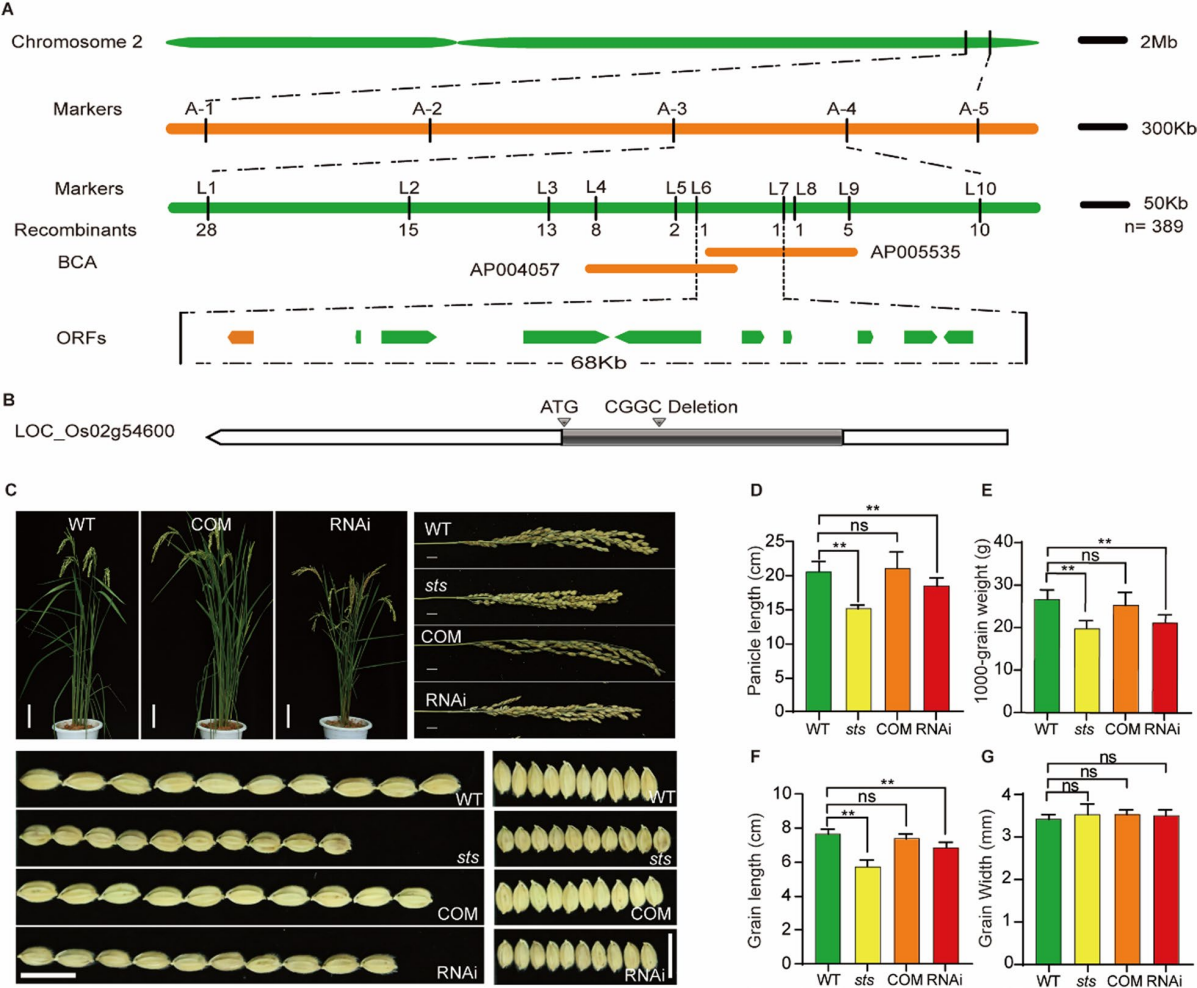
Map-based cloning and characterization of transgenic lines of *OsSTS*. **A** Map-based cloning of *OsSTS*. *OsSTS* was pinpointed in a 68-kb genomic region between molecular markers L6 and L7 on chromosome 2, which contained ten candidate genes. **B** Structure of the *OsSTS* gene. Orange boxes represent exon. The red arrow indicates four base-deletion at the exon of LOC_Os02g54600 that result in a premature translation in *sts*. **C** Phenotypes of wild-type, *sts*, complementation (COM), and RNAi at the adult stage, bar = 10 cm. Comparative observation on panicles and grain size in transgenics, bar = 1 cm. **D–G** Statistical data of the panicle length, 1000-grain weight, grain length, and grain width in transgenics. Data are shown as mean ± SD (n = 15), **P < 0.01, Student’s t-test

To map the *OsSTS* gene, an F1 segregation population was developed by crossing *sts* and the indica cultivar Dular. All F1 plants showed the normal phenotype, similar to that of the wild-type, and the normal to the mutant phenotypes fit the Mendelian segregation ratio (3:1) in the F2 progenies, suggesting that the mutant phenotype was controlled by a single recessive gene. We mapped the mutant locus between SSR markers A-1 and A-5 on chromosome 2 by using 116 F2 mutant individuals. Then using ten SSR/single-nucleotide polymorphism (SNP) markers, the *sts* locus was finally mapped to the region within a region of 68 kb between the markers L6 and L7 (Fig. 2A; Additional file 2: Table S1). According to the Rice Genome Annotation Project (http://rice.plantbiology.msu.edu accessed on 24 March 2021) database, sequencing analysis identified that one of the predicted genes (LOC_Os02g54600) had a 4-bp deletion (CGGC deletion), which caused a frameshift mutation and early termination of transcription (Fig. 2B). Previous studies have shown that the *OsSTS* gene is an allele of the *SMG1* gene, which controls grain type in rice, and encodes a mitogen-activated protein kinase 4 (OsMKK4) (Duan et al. 2014).

Although the functions of the *OsMKK4* gene in controlling grain size have been described previously, its phenotype of complementary and RNAi plants has not been reported. To confirm the functions of this gene in transgenic plants, we obtained the complementary plants of *OsSTS* under the *sts* background and the RNAi plants under the wild-type background. Five T_0_ transgenic positive strains of complementary and RNAi transgenic seedlings were selected respectively for further planting, and the T_1_-generation transgenic positive plants were used for target agronomic character statistics. The results showed that the plant height, panicle length, grain length, and 1000-grain weight phenotypes of all complementary transgenic lines were comparable with those of the wild type. In contrast, the phenotypes of RNAi transgenic lines were similar to those of the *sts* (Fig. 2C–G). These results verified that the loss of function in *OsSTS* was the cause of the small grains of the *sts* mutant.

### Expression Pattern of *OsSTS*

**Fig. 3:**
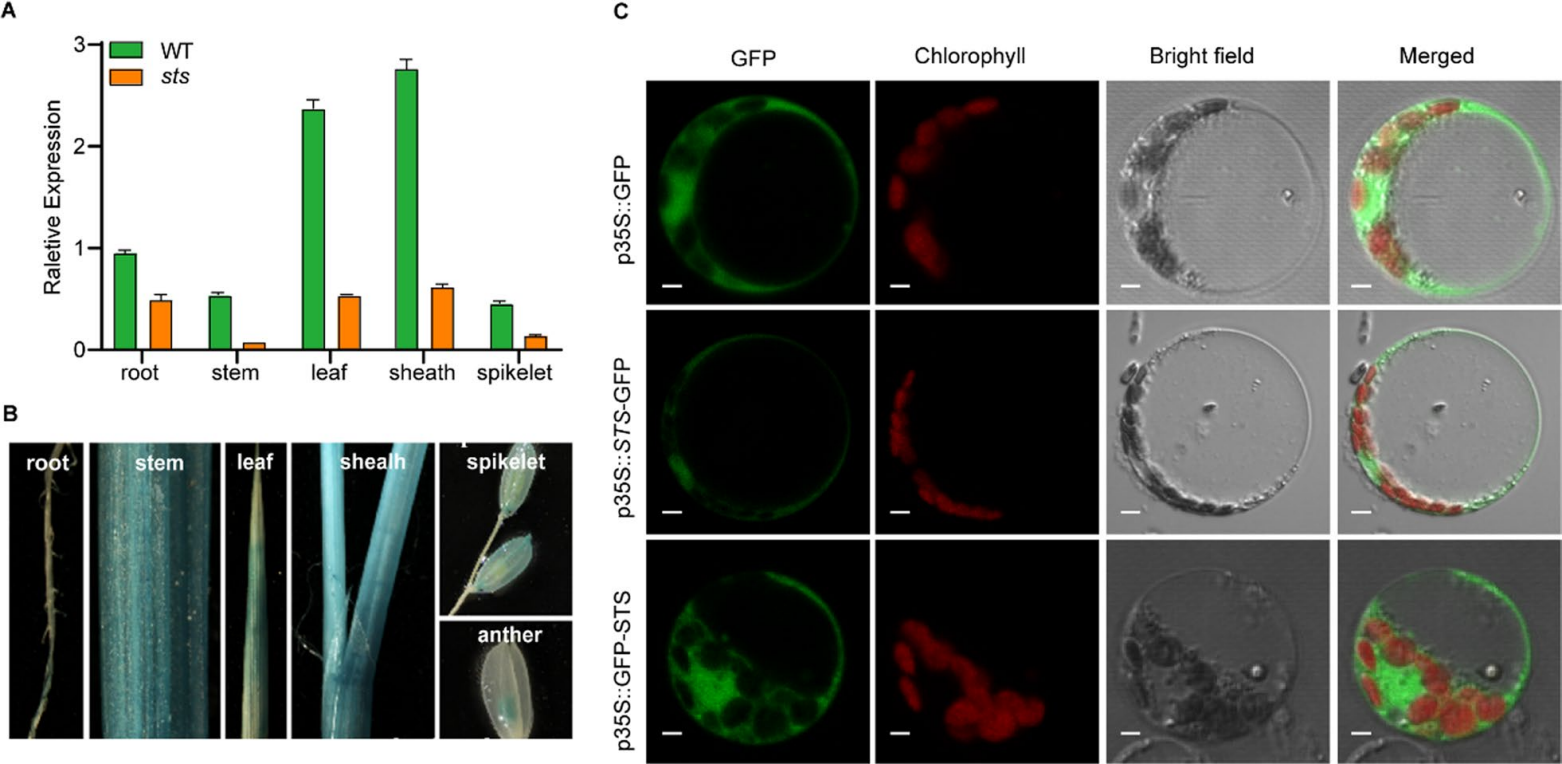
The expression patterns and subcellular localization analysis of *OsSTS*. **A** Transcription analysis of *OsSTS* in different rice tissues by quantitative RT-PCR. Values represent the means ± SD of three biological replicates. **B** GUS staining analysis of *OsSTS* promoter-GUS expression in different rice tissues. root; stem; leaf; sheal; spikelet; anther. **C** Subcellular localization of *OsSTS* in rice protoplasts. Green and red fluorescence shows GFP, and chloroplast autofluorescence, respectively. Bar = 50 μm

The previous results showed that the *OsMKK4* gene appears to be distributed ubiquitously in plant cells. To investigate the expression level of *OsSTS*, we first analyzed the spatial and temporal expression of *OsSTS* in various parts of the rice plant, comparing the wild type to the *sts* mutant. Real-time quantitative PCR (RT-qPCR) results showed that the *OsSTS* gene was expressed in all tissues of rice, with the highest expression in the leaf sheath and the lowest expression in the stem and spikelet (Fig. 3A; Additional file 2: Table S2). To further analyze the spatial expression of *OsSTS* in more detail, we generated transgenic rice plants in which the expression of β-glucuronidase (GUS) was driven by the 2579‐bp promoter region of *OsSTS*. Staining for GUS revealed GUS activity in all the tissues examined, which is consistent with the spatial expression from the qRT‐PCR analysis (Fig. 3B). We further investigated the subcellular localization of OsSTS in order to analyze the effect of the fusion of GFP protein on the N terminal and C terminal of *OsSTS*. The GFP-STS and STS-GFP fused proteins were constructed and introduced into rice protoplasts. Fluorescence microscopy analysis showed that the GFP-STS and STS-GFP fused protein showed the main distribution within the nucleus, cytoplasm, and membrane of rice cells (Fig. 3C), which is almost identical to those of the control of 35S-GFP. Thus, the OsSTS protein may be mainly distributed in the nucleus, cell membrane, and cytoplasm.

### Loss-of-Function of *OsSTS* Enhances Salt Tolerance

**Fig. 4:**
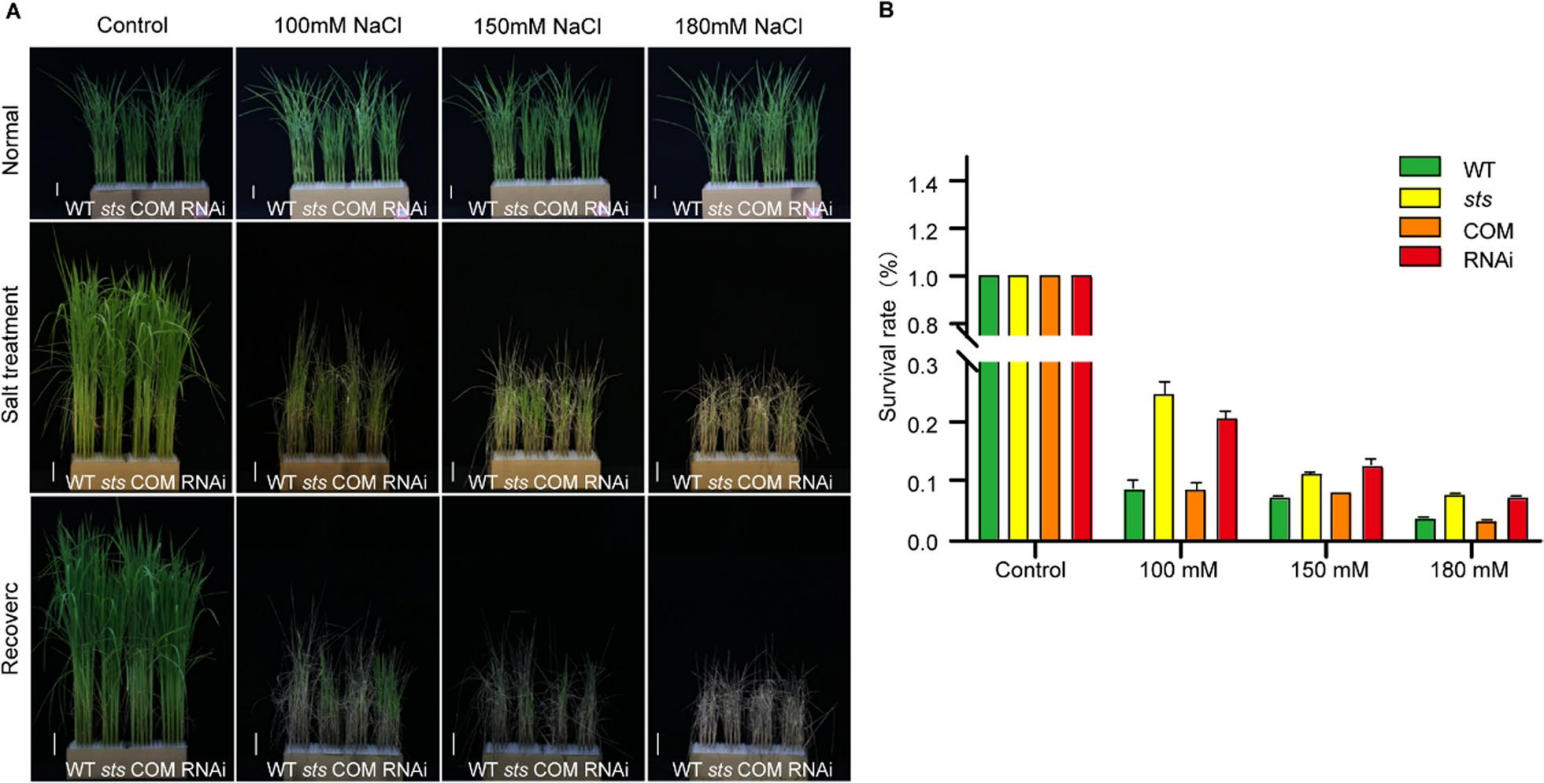
*OsSTS* loss‐of‐function enhances salt tolerance. **A** NaCl stress treatment of wild-type, *sts*, COM, and RNAi. Twenty-day-old plants were treated with different salt concentrations (100 mM NaCl, 150 mM NaCl, and 180 mM NaCl) for two weeks and then recovered as indicated. **B** The survival rate of rice seedlings was recorded with different salt treatments after two weeks of treatment and 10 days of recovery. Experiments were repeated at least three times. Data are shown as mean ± SD

It has been reported that *OsMKK4* participates in disease resistance and the control of grain size and weight through the OsMKKK10-OsMKK4-OsMAPK6 signaling pathway in rice (Kishi-Kaboshi et al. 2010; Xu et al. 2018). Kumar et al. (2008) revealed that the expression level of the *MKK4* gene in indica rice varieties was up-regulated to varying degrees within 12 h of salt treatment, suggesting that specific MAPK cascades act as a junction for crosstalk between different signaling pathways, allowing for the transduction of different signals. However, the mechanism by which the plant MAPK cascade is involved in salt tolerance remains poorly understood. To explore whether the function of *OsSTS* was related to the rice salt stress response, the responses to salt stress were investigated with the seedlings of wild-type and *sts*, COM, and STS-RNAi transgenic plants under different salt concentrations (0mM, 100mM, 150mM, and 180mM). As shown in Fig. 4A, when 20-d seedlings were treated with salt for two weeks with another 10-day recovery (watered without salt), the majority of the wild-type leaves were more wilted compared to the *sts* leaves, while the *sts*-COM plants were similar to the wild-type plants. Conversely, the STS-RNAi plants were more robust than the wild-type plants (Fig. 4A). In addition, the survival rates of the *sts* and STS-RNAi rice seedlings were higher than those of the wild-type plants and STS-COM under three different salt concentrations (Fig. 4B). These results reveal that the functional loss of *OsSTS* showed apparent salt tolerance.

### Destruction of *OsSTS* Increased ROS Clearance in Rice Seedlings

Salt stress can disturb the ion balance and water balance of plant cells, resulting in ion toxicity and osmotic stress, which can negatively impact plant growth (Zhao et al. 2022). To determine the role of *OsSTS* in rice's response to salinity stress, we conducted LiCl and PEG treatment experiments on WT and *sts* plants. Results from the LiCl treatment experiments showed that the shoot and primary root lengths of *sts* were consistent with the WT before and after treatment, implying that *sts* were not affected by ion toxicity (Additional file 1: Fig. S1A–C). Additionally, the shoot and primary root lengths of *sts* were increased significantly under 15% PEG treatment, suggesting that the disruption of *OsSTS* might have some impact on rice osmotic stress tolerance (Additional file 1: Fig. S1D, E). These results prompt us to further explore the reasons behind the increased salt tolerance of *sts*.

**Fig. 5:**
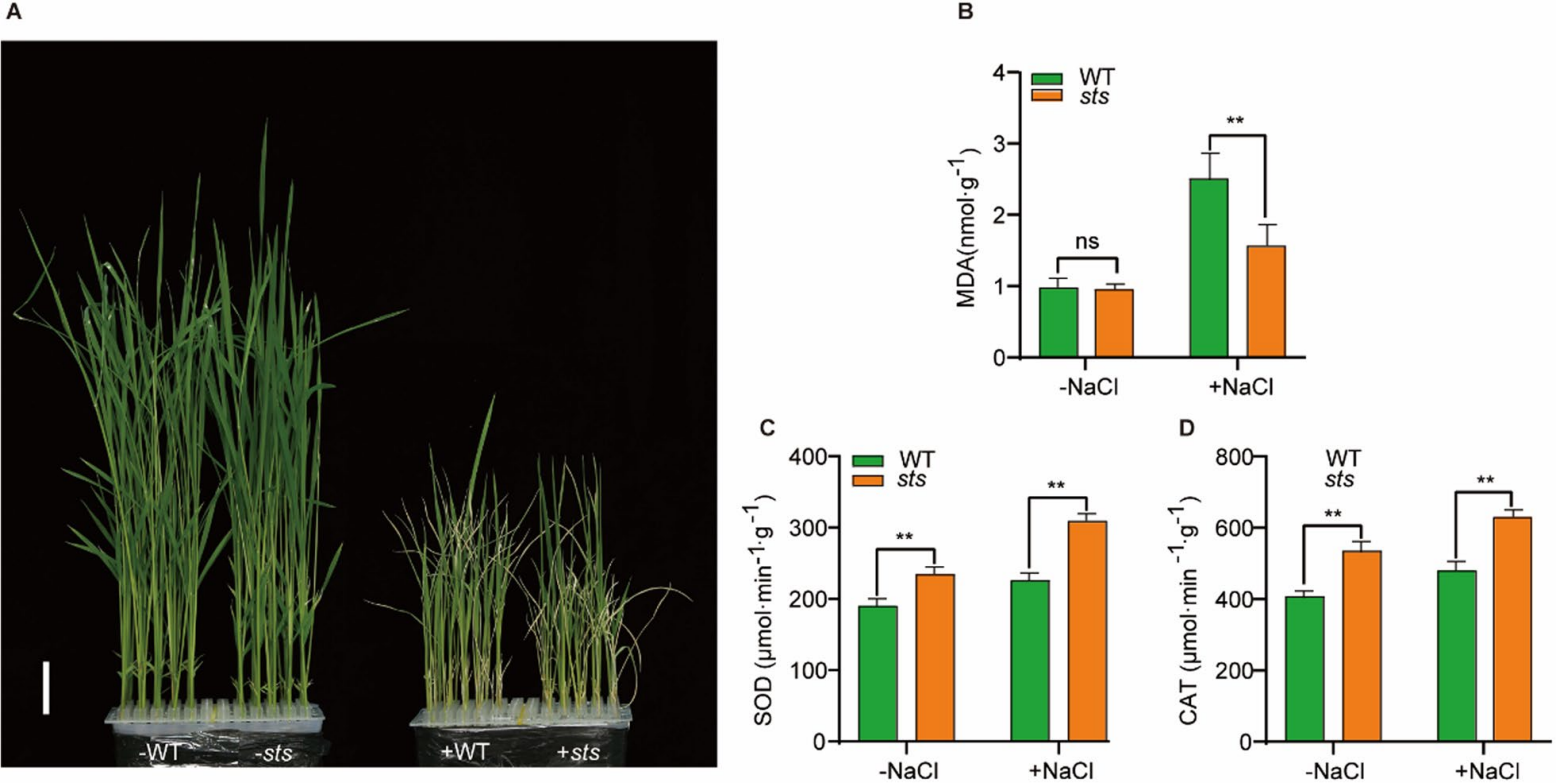
Disruption of *OsSTS* increased ROS clearance. **A** Twenty-day-old plants of WT and *sts* were treated with 150 mM NaCl concentrations for 10 days. -, control treatment; + , NaCl treatment. Bar = 2cm. **B–D** MDA content, SOD activity, CAT activity in leaves of ZH11 and *sts* under normal and salt treatment. − NaCl, control; + NaCl, salt stress. Data are shown as mean ± SD (n = 3). **P < 0.01, Student’s t-test

Additionally, salt stress induces the accumulation of ROS by altering the homeostasis of cellular ROS levels, which leads to oxidative stress-induced toxic effects on the plant (Yang and Guo 2018). To further explore whether the function of *OsSTS* was related to the rice salt stress response through the ROS scavenging system, we analyzed the oxidative damage in WT and *sts* seedlings under salt treatment. The results revealed that MDA content in WT seedlings was markedly higher than that in *sts* seedlings under salt treatment, suggesting that *sts* seedlings experienced less ROS damage (Fig. 5A, B). After 10 days of salt stress, the SOD and CAT contents in both the WT and *sts* increased by 1.18 and 1.31 times, respectively, compared to before treatment (Fig. 5C, D). These results suggested that the disruption of *OsSTS* has a positive role in improving plant salt tolerance, which may be related to the reduction in ROS levels caused by effective ROS clearance.

### *OsSTS* Regulates Global Gene Responses to Salt Stress

**Fig. 6:**
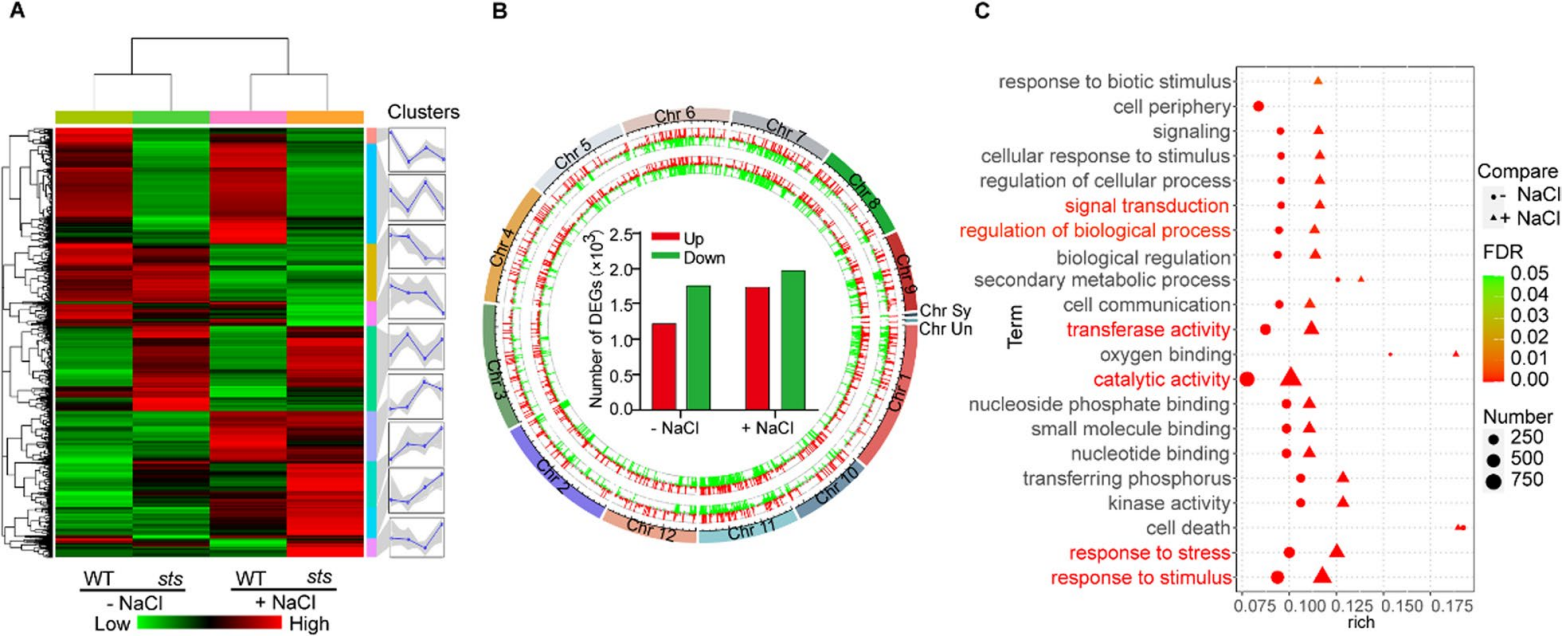
Global transcriptome analysis of *OsSTS*-dependent genes associated with salt stress in rice. **A** Expression pattern of responsive DEGs and clusters upon response to NaCl treatment. The gray shaded markings indicate the corresponding relationship between the left and right panels. B DEGs detected were respectively distributed on 12 chromosomes. *OsSTS* resulted in global changes compared with the WT, both with and without salt stress. **C** Gene ontology (GO) enrichment analysis of DEGs between WT and *sts* both with and without salt stress (*P* ≤ 0.05)

To further elucidate the potential molecular mechanisms by which *OsSTS* is involved in the transcriptional regulation of the rice salt response, we studied the global impression profiles of *OsSTS*-dependent gene expression in response to salt stress by analyzing the transcriptome of WT and *sts* that were treated under normal and salt stress conditions (150 mM NaCl). Through this method, we found that 7726 differentially expressed genes (DEGs) showed differential expression patterns with or without salt stress. Meanwhile, these DEGs (–NaCl and +NaCl conditions) were further clustered into nine clusters (SR1-9) based on their expression patterns in the WT and mutants (Fig. 6A; Additional file 3: Table S3). And then, a total of 6670 DEGs detected were respectively distributed on 12 chromosomes, compared with the WT both with and without salt stress (Fig. 6B). Under normal conditions (-NaCl conditions), we identified 1212 upregulated genes and 1756 downregulated genes in *sts* compared with WT, while 3702 DEGs (1730 upregulated and 1972 downregulated) were detected under salt stress conditions (Fig. 6B). Gene ontology (GO) classifications further revealed that the top enriched GO terms were related to the catalytic activity (GO:0003824), response to stimulus (GO:0050896), response to stress (GO:0006950), transferase activity (GO:0016740), regulation of biological process (GO:0050789), and signal transduction (GO:0007165) under both normal and salt stress conditions (Fig. 6C; Additional file 4: Table S4). Moreover, 1109 DEGs (-NaCl) and 1434 DEGs (+ NaCl) were respectively associated with molecular function, and 1199 DEGs (–NaCl) and 1489 DEGs(+NaCl) respectively associated with the biological process were also revealed, suggesting that salt stress also influenced molecular function and biological process in the mutants (Fig. 6C; Additional file 4: Table S4). A Kyoto Encyclopedia of Genes and Genomes (KEGG) pathway enrichment analysis showed a large number of the DEGs were enriched in two major signaling pathways related to hormone signaling and the mitogen-activated protein kinase signaling pathway associated with salt stress (Additional file 1: Figure S2A, B; Additional file 2: Additional file 5: Table S5). These results imply that the salt-stress tolerance of *sts* may be related to the hormone signaling pathway.

### Loss-Function of *STS* Also Causes Increased Transcript Levels of ABA Responsive Genes

**Fig. 7:**
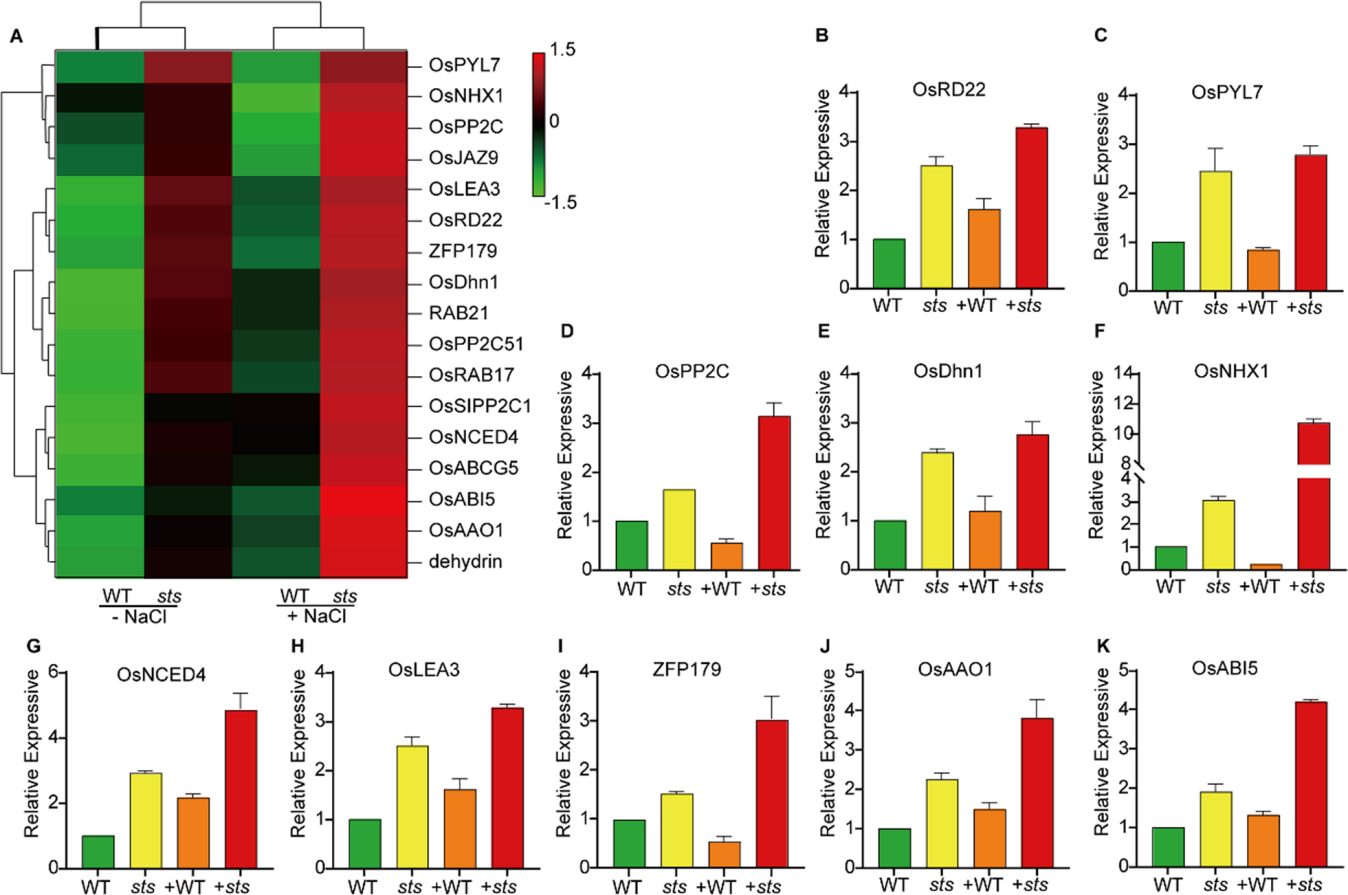
Transcript levels of ABA‐ and salt‐stress‐responsive genes. **A** Transcript levels of ABA‐ and salt‐stress‐responsive genes between WT and *sts* under the two different experimental conditions by RNA-seq. **B–K** Verification of the relative expression in response to different treatments of some *OsSTS* differentially expressed genes by qRT-PCR. The expression level of genes in WT under normal conditions is standardized to ‘1’. Data are given as means ± SD (n = 3)

Our RNA-seq analysis showed significant differences between the wild type and mutant in the MAPK (04016) signal pathway and plant hormone signal transduction (04075) before and after salt treatment. (Additional file 1: Figure S3A, B; Additional file 6: Table S6). To further investigate these pathways, we then chose 17 reported significantly up-regulated differential genes of DEGs related to the MAPK signal pathway and the plant hormone signal transduction for further analysis (Fig. 7A; Additional file 6: Table S6). Previous studies have shown that the expression levels of *OsSIPP2C1*, *OsABI5*, *OsNHX1*, *OsLEA3*, and *ZFP179* genes were affected by high salt and ABA stress. *OsNCED4* and *OsAAO1* are involved in ABA synthesis, *OsPYL7* is an ABA receptor, *OsPP2C* and *OsPP2C51* are involved in ABA response, and signal transduction, *OsRAB17*, *OsRD22*, *dehydrin*, and *RAB21* are involved in ABA response, and *OsABCG5* is involved in ABA transport. And the remaining *OsDhn1* and *OsJAZ9* were involved in salt stress response. We finally randomly selected ten genes from the up-regulated genes after salt treatment for expression analysis by RT-qPCR. As a result, a high correlation was found between the RNA-seq and qRT-PCR results, confirming the accuracy of the RNA-seq data (Fig. 7B–K; Additional file 7: Table S7). To further analyze the role of ABA in salt-induced *OsSTS* expression, we first studied the expression pattern of *OsSTS* under salt treatment by RT-qPCR. After salt treatment, the expression of *OsSTS* in WT was up-regulated within 12 h (Additional file 1: Fig. S4A), especially after 1 h treatment, the expression of *OsSTS* was approximately 2.8 times higher than that in WT under normal treatment, and the expression of *OsSTS* in *sts* was significantly lower than that in WT, indicating that salt treatment induced the expression of OsSTS. (Additional file 1: Fig. S4A; Fig. 3A). Following, we analyzed the expression of *OsSTS* with ABA treatment. The result from RT-qPCR assays showed that the expression of *OsSTS* under exogenous ABA treatment was approximately 3.2 times of the normal treatment after 1 h of ABA treatment, thus indicating that the expression of *OsSTS* was slightly induced by ABA (Additional file 1: Fig. S4B). These findings suggest that the *sts* mutant's salt-stress tolerance is related to the hormone signaling pathway and regulates *OsSTS* expression to affect ABA sensitivity."

### ABA is Critical for STS to Modulate Rice Salt Stress Tolerance

**Fig. 8:**
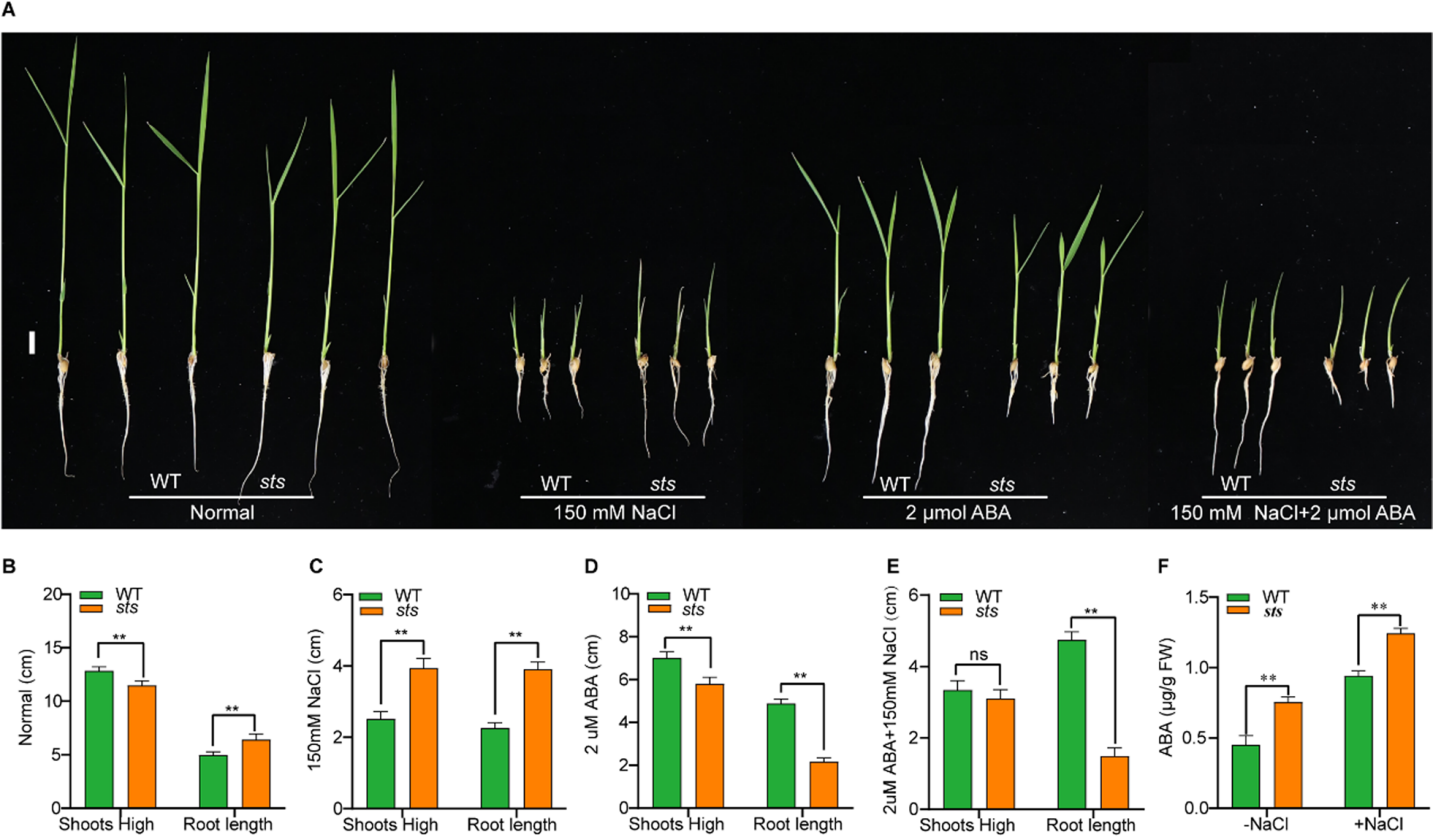
ABA is related to the *OsSTS* function. **A** Phenotypes of wild-type and *sts* under different treatments (Normal; 150 mM NaCl; 2 µM ABA; 150 mM NaCl and 2 µM ABA) at rice seedlings stage, bar = 1 cm. **B–E** Shoots high and root length of wild-type and *sts* under different treatments. Data are shown as mean ± SD (n = 10). **F** ABA content. Data are given as means ± SD (n = 3). **P < 0.01, Student’s t-test

To further analyze the role of the *OsSTS* gene in salt and ABA stress in rice, we examined the length of shoots and primary roots under normal treatment, salt stress treatment, ABA stress treatment, and combined salt and ABA stress conditions. We found that the length of *sts* shoots was more severely inhibited under salt stress conditions compared to WT, while the length of primary roots between WT and *sts* was both inhibited, and the degree of inhibition was lighter than that of the wild type (Fig. 8A–C). Under ABA stress conditions, the length of *sts* roots was more severely inhibited compared to that of WT (Fig. 8A, B, D). At the same time, both the shoot length and root length of *sts* were significantly inhibited under the combined stress of salt and ABA, while the root length of WT had no significant difference from that of normal condition or ABA treatment (Fig. 8A, B, D, E). We speculated that ABA alleviates the inhibitory effect of salt stress on root length in *sts* (Fig. 8). The results of the ABA content measurement of WT and *sts* before and after salt treatment showed that the ABA content of *sts* was significantly higher than that of the wild type with or without salt treatment (Fig. 8F).

## Discussion

Salt stress is a common abiotic stress that affects plant growth and development by mainly disrupting ion homeostasis and inducing osmotic stress (van Zelm et al. 2020). Plants respond to salt stress through various biological processes, including the activation of MAPK pathways, which influence signal transduction in response to biotic and abiotic stresses, hormones, cell division, and developmental processes (Jagodzik et al. 2018; Zhu 2002; Deinlein et al. 2014). OsMKKK10-OsMKK4-OsMAPK6 has been reported to play critical roles in regulating not only grain size but also multiple aspects of growth and development in rice (Xu et al. 2018; Duan et al. 2014). In this study, we demonstrate that *sts*/*osmkk4* not only shares the small and short grains phenotype but also enhances plant salt tolerance, providing a broader functional understanding of salt tolerance in rice.

As an essential determining factor of rice production, grain size has been extensively studied, and several genes regulating grain size have been characterized (Li et al. 2019; Zuo and Li 2014). MAPK signaling pathways conserved signaling mechanisms in eukaryotes have been reported to play important roles in various processes related to plant development (Xu and Zhang 2015). Here, our findings demonstrate that the smaller grain length in *sts* can be recovered by transferring the complementary vector into the *sts*, and the smaller grain length of the RNAi lines was similar to that of the *sts*, suggesting that the allelic mutations of *sts* control grain size, which is consistent with the previous results (Xu et al. 2018; Duan et al. 2014). The phenotypic result of *smg1-1*/*smg1-2* mutants previously studied was significantly decreased compared with that of the SF43 and Nipponbare, respectively, while the primary and secondary branches were slightly increased without significant difference (Xu et al. 2018; Duan et al. 2014). Our results showed that the number of primary and secondary panicle branches in *sts* was significantly increased compared with that in ZH11, and there is no significant change in the *sts* grain width (Fig. 1). These results suggest that the *OsSTS* gene regulates the grain length, and other differences in agronomic traits may be due to the different genetic backgrounds of the gene mutants.

Previous studies showed that *OsMKK4* was involved in regulating cold signal and salinity stress but not in the transduction of drought and heat stress through real-time quantitative PCR analysis (Kumar et al. 2008). Also, the *smg1*/*osmkk4* mutant has been reported as relatively less sensitive to brassinosteroid (BR) and affected the expression of BR biosynthetic genes (Duan et al. 2014). Here, this study showed that the expression of *OsSTS* was significantly increased after 1 h of salt treatment or ABA treatment, indicating that *OsSTS* is not only regulated by salt stress but also by ABA. Therefore, these findings suggested a possible connection between the MAPK cascades and the ABA signal. Notably, our results showed that under salt and ABA stress, the root length of *sts* is inhibited by superposition, while the inhibition effect of salt stress on *STS* root length was relieved. This leads us to ponder whether a suitable concentration of ABA can alleviate the damage of salt stress on rice plants in different rice varieties planted under salt stress or in saline-alkali soil. Of course, further experiments are needed to prove this conjecture.

The overaccumulation of ROS is a primary response of plants to salt stress, which can disrupt biological macromolecules and cause toxic effects on cells (Cui et al. 2021; Mittler 2017). The steady-state levels of ROS can be influenced by ROS clearance and production mechanisms (Mittler 2017). In our study, we observed that many genes related to the GO terms ‘catalytic activity,’ ‘response to stress,’ and ‘response to stimulus’ were either upregulated or downregulated in rice seedlings under salt stress, suggesting that the ROS content in *sts* may have changed. To investigate this, we measured the MDA content, which can represent the degree of oxidative damage in cells, and found that it was at a low level in *sts* seedlings under salt stress, indicating that there was only slight ROS damage present in these seedlings. Guard cells containing both enzymatic and nonenzymatic machinery can maintain ROS homeostasis during ABA signaling via antioxidant enzymes (Postiglione and Muday 2020). SOD and CAT are crucial antioxidant enzymes that play critical roles in enhancing the tolerance of the plant to environmental stresses by scavenging ROS (Wang et al. 2022c; Meng et al. 2020). Our results showed that the activities of CAT and SOD were significantly enhanced in *sts* seedlings before and after salt treatment, thus indicating that the increased salt tolerance of *sts* may be due to enhanced ROS scavenging ability during ABA signaling.

Studies showed that MAPK signaling could occur in response to plant cell differentiation and development, maturation, hormone signal transduction, and immune processes via phosphorylation of multiple transcription factors and other signaling pathway components (Wang et al. 2022b; de Zelicourt et al. 2016; Danquah et al. 2014). Meanwhile, MAPK protein kinases also affect intracellular responses and functions under biotic and abiotic stresses (Danquah et al. 2014; Jagodzik et al. 2018). In our study, RNA-seq results revealed that many genes related to the KEGG terms ‘MAPK signaling pathway’ and ‘Plant hormone signal transduction’ were found to be upregulated or downregulated in rice seedlings under salt stress, suggesting that the crosstalk mechanisms in *sts* seedlings may have existed. The crosstalk mechanisms were valued highly between MAPK cascades and plant hormones in plants, mainly including AUX, ABA, ETH, BR, etc. (Jagodzik et al. 2018). Our results showed that the transcript levels of some ABA-responsive genes were increased (Fig. 7). The ABA-signaling pathway is central to abiotic stress responses in plants, triggering major changes in plant physiology (Huang et al. 2021; Zhang et al. 2022), and the biosynthesis and transport of ABA in a plant can adapt physiological processes to the prevailing stress conditions (de Zelicourt et al. 2016). The analysis of gene transcript level showed that the genes related to ABA synthesis and response were found to be upregulated, such as *OsNCED4* (Zhu et al. 2009), *OsPYL7* (He et al. 2014; Kim et al. 2012; Tian et al. 2015), *OsSIPP2C1* (Singh et al. 2015), *OsPP2C51* (Bhatnagar et al. 2017). Therefore, we speculated that the disruption of the *sts* function increased ABA synthesis. This research enriches our understanding of the link between the MAPK signaling pathway and Plant hormone signal transduction in plants under abiotic stresses.

## Materials and Methods

### Plant Materials and Growth Conditions

The *sts* mutant was isolated from the mutant rice library of *Oryza sativa* L. ssp. *japonica* cultivar Zhonghua11 (ZH11) irradiated with ^60^Co-γ in this study. All of the transgenic plants we obtained and their offspring were grown in the greenhouse under continuous temperature (30 °C at 16 h light and 28°C at 8 h dark) in winter. During the planting season, all rice plants were cultivated in the paddy fields of the China National Rice Research Institute in Hangzhou.

### Map-Based Cloning of *OsSTS*

For map-based cloning of *OsSTS*, 389 F2 plants with mutant-like phenotypes were generated from the cross of ZH11 with the indica rice cultivar Dular. The locus was first mapped to an interval between the two markers A-3 and A-4 (Additional file 2: Table S1) on the long arm of chromosome 2, then further narrowed down to a 68-kb DNA region using newly developed markers based on the nucleotide polymorphisms in the corresponding regions between the cultivars ZH11 and Dular. Gene prediction was performed using the publicly available rice database, Rice Genome Annotation Project (http://rice.plantbiology.msu.edu/index.shtml).

### Complementation Assay and RNAi

For complementation of the *sts* mutation, a 5460-bp genomic DNA fragment from ZH11 containing the entire *OsSTS* coding region, along with 2579-bp upstream and 1771-bp downstream of the gene, was amplified with high-fidelity enzyme KOD Plus (Toyobo, Tokyo, Japan) and inserted into the binary vector pCAMBIA1300 by homologous recombination. The resulting construct pCAMBIA1300-*STS* was transformed into *sts* calli to obtain complementary transgenic plants. To generate the *STS*-RNAi lines, two 266-bp fragments of *STS*-RNAi cDNA were amplified and inserted downstream of the Ubi promoter in vector pTCK303. The constructed plasmid was transferred into ZH11 by the Agrobacterium-mediated transformation method. Gene-specific primers are listed in Additional file 2: Table S1.

### RNA Extraction and Quantitative Real-Time PCR (qRT-PCR)

RNA from different tissues was extracted using the Total RNA Miniprep kit (Axygen, Hangzhou, China) according to the manufacturer’s instructions. cDNA was synthesized with the ReverTra Ace qPCR-RT kit (Toyobo, Osaka, Japan), and then the RT-PCR experiment was performed using the SYBR Premix Ex Taq (Takara, Kusatsu, Japan), and gene-specific primers on a CFX96 Touch Real-time PCR Detection System. Three biological replicates were performed for all experiments. Rice ACTIN1 was used as the internal control for all analyses. Primers used in this experiment are listed in Additional file 2: Table S2.

### Histological GUS Assay

The promoter of *OsSTS* (2579‐bp upstream of the start codon) was cloned into the binary vector pCAMBIA1305.1 to generate pCAMBIA1305‐*PRO STS*::GUS vector (*PRO STS*::GUS). The recombinant vector was then transformed into calli of ZH11 to obtain transgenic plants. For GUS staining, different tissues of transgenic plants were collected from *PRO STS*::GUS transgenic plants, followed by incubation in GUS staining buffer for 12 h at 37 °C (Wang et al. 2022a). The samples were dehydrated in 75% (v/v) ethanol to clear the chlorophyll, then scanned using a Microtek Scan Maker i800 plus.

### Subcellular Localization of *OsSTS*

To investigate the subcellular localization of *OsSTS*, the full-length *OsSTS* cDNA without the stop codon was amplified with primers STS-GFP-F and STS-GFP-R (Additional file 2: Table S1). The resulting fragment was inserted into the transient expression vector 163-35S::GFP to produce the 163-35S::STS‐GFP and the 163-35S::GFP‐STS constructs. rice protoplasts were then co-transfected with p35S::STS‐GFP/p35S::GFP‐STS and control vector (163-35S::GFP) according to the protocols described previously (Nelson et al. 2007; Cui et al. 2021). The fluorescence signals were observed using a Zeiss LSM700 laser scanning confocal microscope.

### RNA-seq and Data Analysis

ZH11 and *sts* plants were cultivated for 21 d under normal conditions or for 14 d under normal conditions, and 7 d under salt treatment (1% NaCl). Three biological replicates (each consisting of pooled leaves) were collected and then snap-frozen in liquid nitrogen for total RNA extraction. The 12 libraries were constructed and sequenced on an Illumina HiSeq platform. HTSeq was used to estimate the original expression of the gene. DEGs were identified using significant *P*-value < 0.05 and expression difference multiple |log2FoldChange|> 1. GO enrichment analysis for the DEGs was implemented with topGO (the standard of significant enrichment is *P*-value < 0.05). ClusterProfiler (3.4.4) software was used to carry out the enrichment analysis of the KEGG pathway of differential genes.

### Treatments Using NaCl, ABA, LiCl, and PEG

To study the salt stress tolerance of rice seedlings, 20-d-old seedlings grown in a 96-well plate under normal conditions were transferred to Yoshida’s culture solution containing 0, 100 mM NaCl, 150 mM NaCl, and 180 mM NaCl. Two weeks after transplantation, the rice seedlings were removed from the NaCl culture solution and grown under normal conditions. The survival rate of rice seedlings and counted after a 10-day recovery period, and the criterion for death was the absence of green shoots.

For salt sensitivity analyses of the rice, seeds were germinated in water and were then planted on Yoshida’s culture solution with or without 150 mM NaCl for 10 days. To analyze the sensitivity of *sts* to exogenous ABA, the rice seeds were germinated and then planted on Yoshida’s culture solution or Yoshida’s culture solution supplemented with 2 µM ABA for another 10 days. To study the role of STS in rice ion stress tolerance, the germinated rice seeds were grown on 1/2 MS with or without containing 18 mM LiCl for another 10 days. To analyze the effect of *sts* on rice osmotic tolerance, germinated rice seeds were grown on Yoshida’s culture solution or 15% (w/v) PEG (polyethylene glycol 6000) contained in Yoshida’s culture solution for another 10 days. To investigate the effect of ABA on *OsSTS*-regulated salt tolerance, the germinated rice seeds were planted on Yoshida’s culture solution with or without 150 mM of NaCl and 2 µM of ABA for 10 days. The length of shoots and the primary roots were measured after 10 days of salt treatment, and photos of the seedling phenotype were observed and taken.

### Determination of Stress-Related Physiological Index

The appropriate kits for measuring SOD, CAT enzyme activities, and MDA content of rice seedlings were purchased from Geruisi following the manufacturer’s instructions with three biological replicates per sample (http://www.geruisi-bio.com/).

### Measurement of ABA Content

The endogenous ABA content was measured using the HPLC method (High-Performance Liquid Chromatography). The rice seedlings (0.1g) snap-frozen were ground to a fine powder with liquid nitrogen. The powder was extracted following the manufacturer’s instructions. ABA content was quantified using a Wufeng LC-100 system. The experiments were performed with three biological replicates per sample.

### Quantification and Statistical Analysis

Quantification analyses were performed on all the measurements in GraphPad Prism 9. All statistical analyses were performed by using a student’s t-test and one-way analysis of variance (ANOVA) among treatments. The experiments were conducted three times at least and designed with a randomized complete block.

### Supplementary Information

**Additional file 1: Fig S1**. Disruption of *OsSTS* affect osmotic stress tolerance of rice. **Fig S2**. KEGG pathway enrichment analysis of *OsSTS*-dependent genes associated with salt stress in rice. **Fig S3**. DEGs associated with salt stress were enriched in MAPK signal pathway and plant hormone signal transduction in rice. **Fig S4**. Salt-induced expression of *OsSTS* affects rice sensitivity to ABA.

**Additional file 2**: **Table S1**. Primers used in this study. **Table S2.** qPCR primers used in this study.

**Additional file 3: Table S3.** A list of responsive DEGs and clusters upon response to NaCl treatment.

**Additional file 4: Table S4.** A list of gene ontology (GO) enrichment analysis of DEGs between WT and sts both with and without salt stress.

**Additional file 5: Table S5.** A list of KEGG enrichment analysis of DEGs between WT and sts both with and without salt stress.

**Additional file 6: Table S6.** A list of DEGs enriched in MAPK signal pathway and plant hormone signal transduction under salt stress.

**Additional file 7: Table S7.** A list of ABA‐ and salt‐stress‐responsive genes between WT and sts under the two different experimental conditions by RNA-seq.

## Data Availability

All data generated or analysed during this study are included in this published article and its supplementary information files.

## Abbreviations

*sts*: Salt-tolerant and small grains
ROS: Reactive oxygen species
MAPK: The Mitogen-activated protein kinase
AUX: Auxin
ABA: Abscisic acid
ETH: Ethylene
BR: Brassinosteroids
CAT: Catalase
SOD: Superoxide dismutase
SNP: Single-nucleotide polymorphism
COM: Complementation
GUS: β-Glucuronidase
DEGs: Differentially expressed genes
GO: Gene ontology
KEGG: Kyoto encyclopedia of genes and genomes
HPLC: High-performance liquid chromatography
ANOVA: Analysis of variance
