# Supplementary material for: OsSTS, a Novel Allele of Mitogen-Activated Protein Kinase Kinase 4 (OsMKK4), Controls Grain Size and Salt Tolerance in Rice
Source: Rice (N Y). 2023 Oct 24;16:47. doi: 10.1186/s12284-023-00663-y (PMC10597928; doi:10.1186/s12284-023-00663-y)
Supplement: Supplementary file 1 — Additional file 1: Fig S1. Disruption of OsSTS affect osmotic stress tolerance of rice. Fig S2. KEGG pathway enrichment analysis of OsSTS-dependent genes associated with salt stress in rice. Fig S3. DEGs associated with salt stress were enriched in MAPK signal pathway and plant hormone signal transduction in rice. Fig S4. Salt-induced expression of OsSTS affects rice sensitivity to ABA. [file 12284_2023_663_MOESM1_ESM.docx]

**Supplementary Material**

***OsSTS*, a novel allele of Mitogen activated protein Kinase Kinase 4 (*OsMKK4*) controls grain size and salt tolerance in rice**

Authors:

Jianguo Liu ^1,2 †^, Lan Shen ^2†^, Longbiao Guo^2^, Guangheng Zhang^2^, Zhenyu Gao^2^, Li Zhu^2^, Jiang Hu^2^, Guojun Dong^2^, Deyong Ren^2^, Qiang Zhang^2^, Qing Li^2^, Dali Zeng^4*^, Changjie Yan^3*^, Qian Qian^2*^

^1^ Rice Research Institute, Shenyang agricultural University, Shenyang 110866, China

^2^ State Key Laboratory of Rice Biology and Breeding, China National Rice Research Institute, Chinese Academy of Agricultural Sciences, Hangzhou 311401, China

^3^ Jiangsu Co‐Innovation Center for Modern Production Technology of Grain Crops/Agricultural College, Yangzhou University,Yangzhou 225009, China

^4^ The Key Laboratory for Quality Improvement of Agricultural Products of Zhejiang Province, College of Advanced Agricultural Sciences, Zhejiang A & F University, Hangzhou 311300, China

^*^Author for correspondence: Dali Zeng([dalizeng@126.com](mailto:dalizeng@126.com)), Changjie Yan ([cjyan@yzu.edu.cn](mailto:cjyan@yzu.edu.cn)), Qian Qian ([qianqian188@hotmail.com](mailto:qianqian188@hotmail.com)).

^†^ These authors contributed equally to this work.


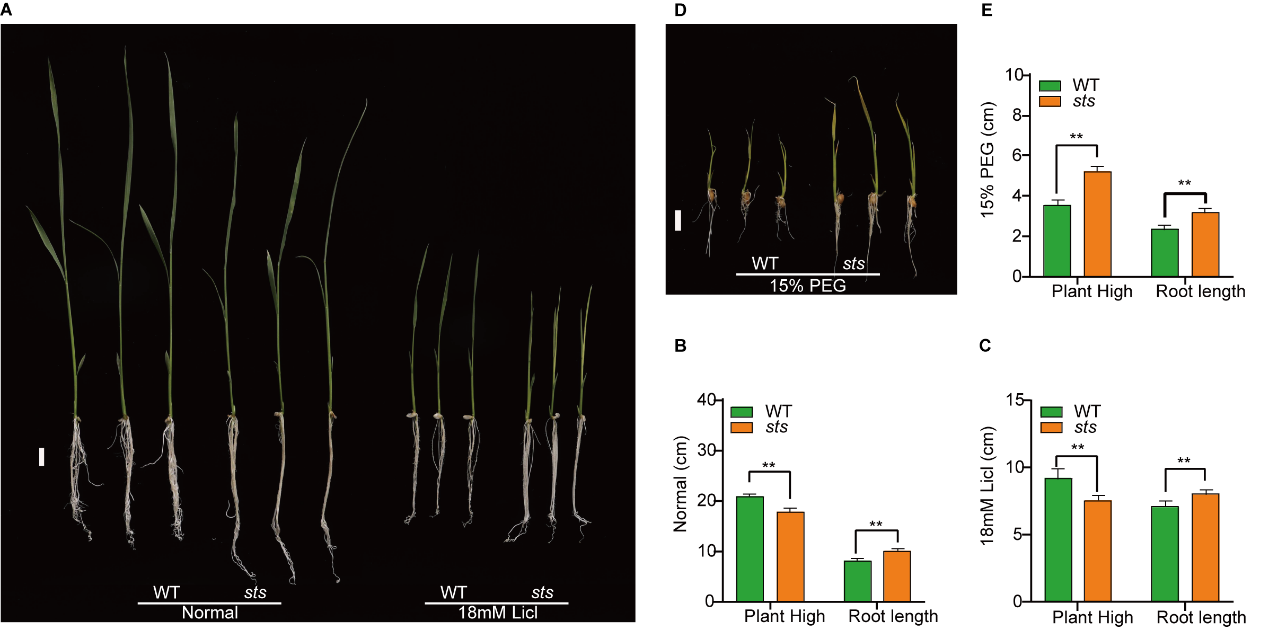


**Fig. S1** Disruption of *OsSTS* increased rice salt tolerance. **A, D** Phenotypes of wild-type and *sts* under different treatments (Normal; 18 mM LiCl; 15% PEG treatment) at rice seedlings stage, bar = 1 cm. **B, C, E** Shoots high and root length of wild-type and *sts* under different treatments. Data are shown as mean ± SD (n = 10). **, P < 0.01, Student’s t-test.

**
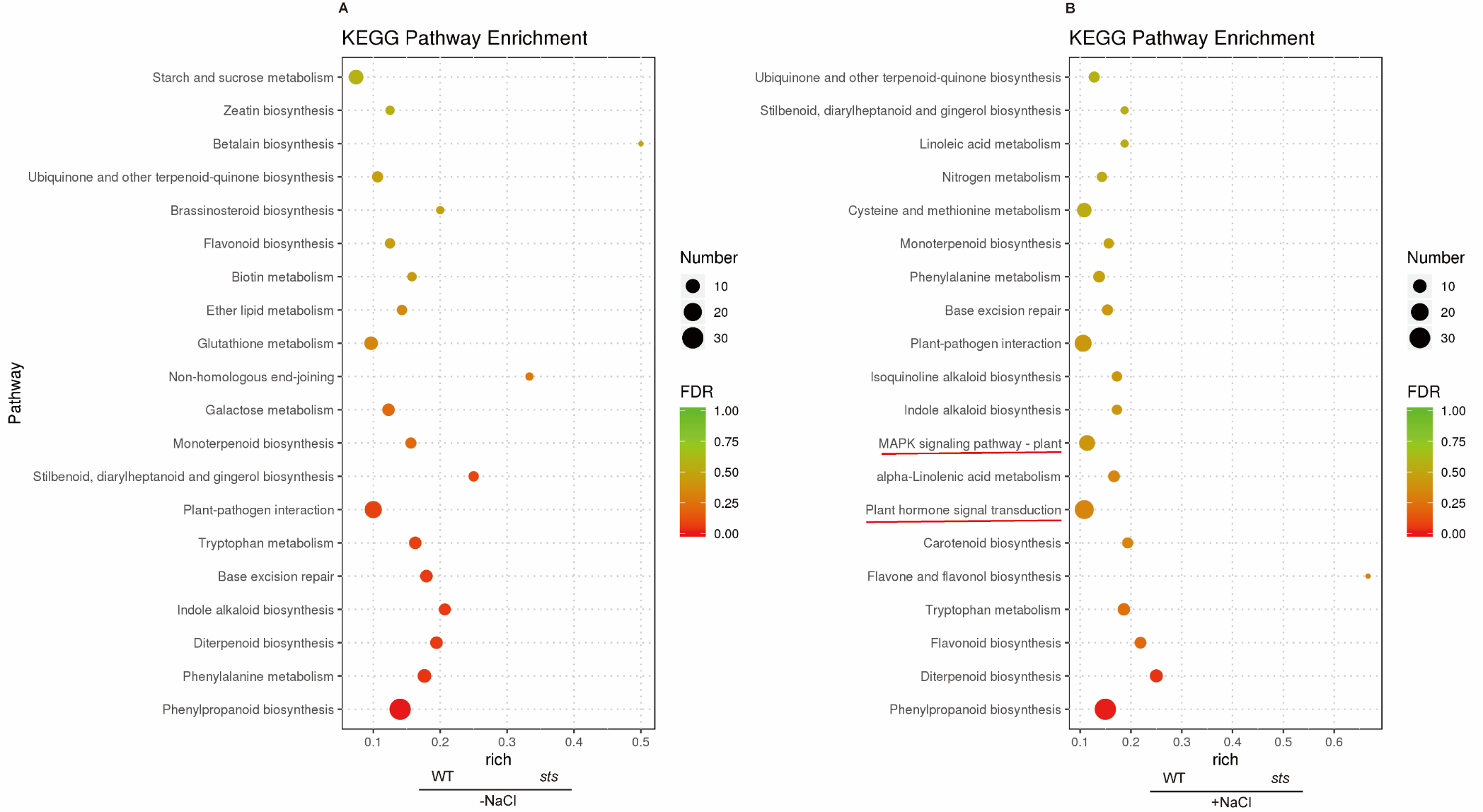
**

**Fig. S2** KEGG pathway enrichment analysis of *OsSTS*-dependent genes associated with salt stress in rice. **A, B** KEGG enrichment analysis of DEGs between WT and *sts* both with and without salt stress (P ≤ 0.05)


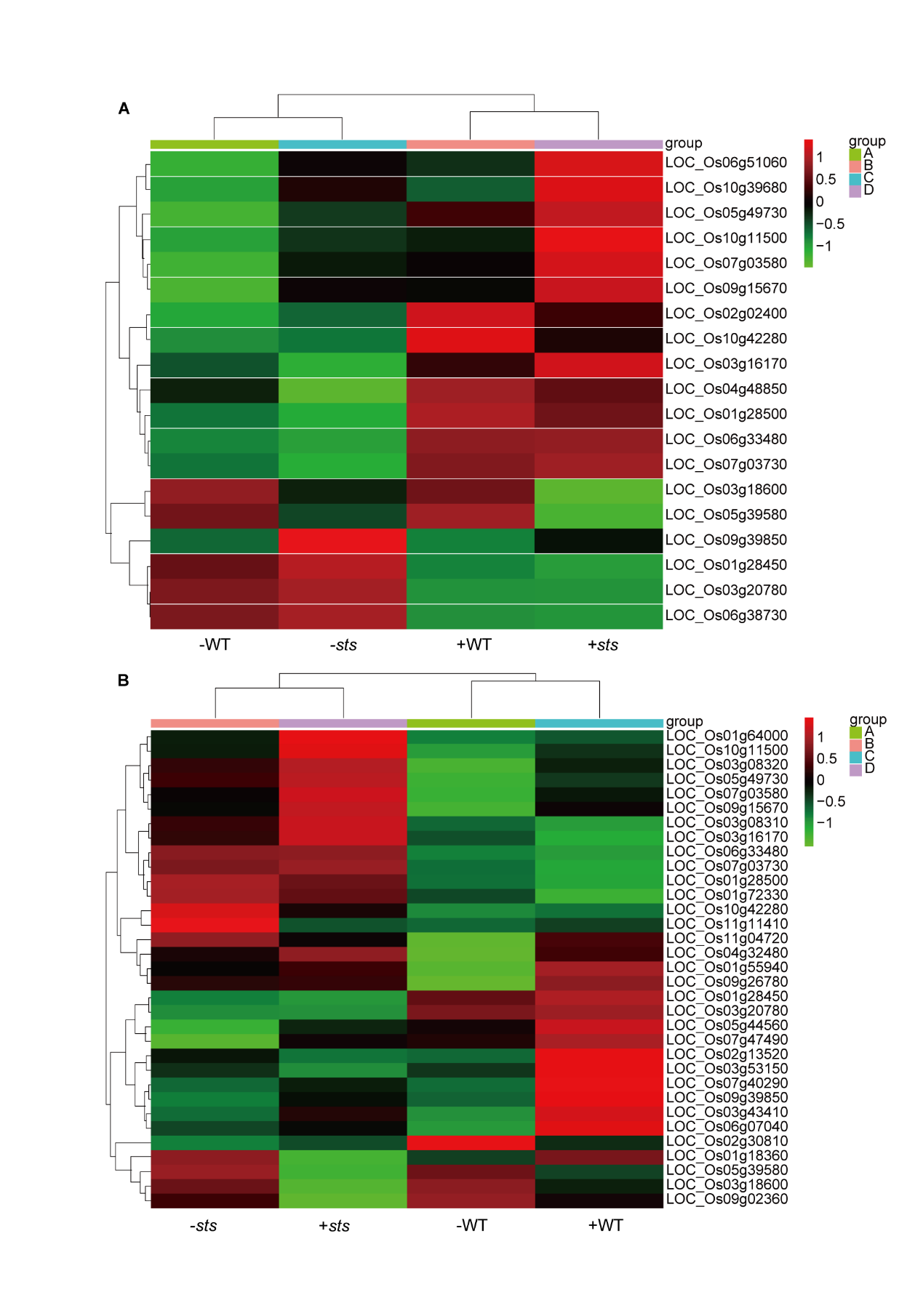


**Fig. S3** DEGs associated with salt stress were enriched in MAPK signal pathway and plant hormone signal transduction in rice. **A** MAPK signal pathway. **B** Plant hormone signal transduction. (P ≤ 0.05)


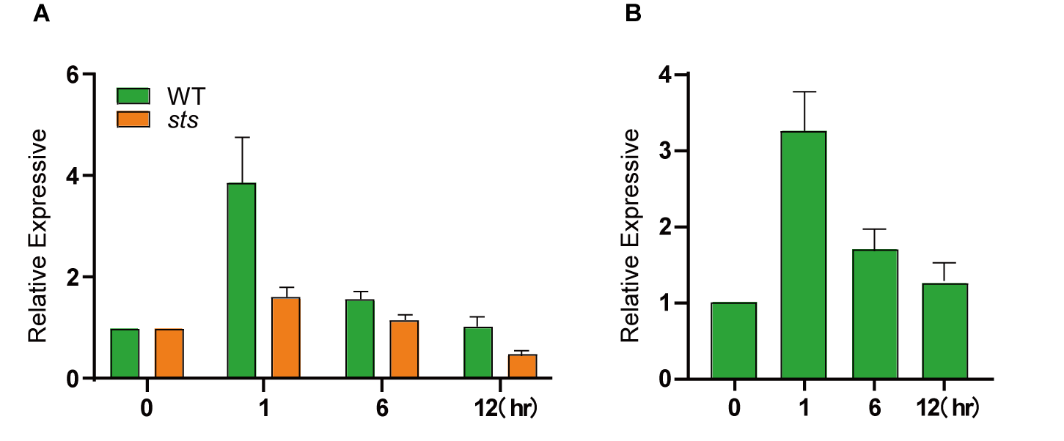


**Fig. S4** Salt-induced expression of *OsSTS* affects rice sensitivity to ABA. **A** Expression pattern of *OsSTS* in WT and *sts* under salt treatment. The *OsSTS* expression level under normal conditions (0 h) is standardized to ‘1’. **B** Expression pattern of *OsSTS* under ABA treatment. Under normal conditions, the *OsSTS* expression level in WT is standardized to ‘1’. Shown are mean ± SD from three biological replicates.
